# Pathogenicity in Chicken Anemia Virus with *Eimeria tenella*: Concurrent Co-Infection and Secondary *Eimeria tenella* Infection

**DOI:** 10.3390/microorganisms13071676

**Published:** 2025-07-16

**Authors:** Hsyang-Hsun Chung, Suttitas Tongkamsai, Ming-Chu Cheng, Yi-Lun Tsai, Meng-Shiou Lee, Yi-Yang Lien, Ya-Mei Chen

**Affiliations:** 1 Department of Veterinary Medicine, College of Veterinary Medicine, National Pingtung University of Science and Technology, Pingtung 912301, Taiwanyltsai@mail.npust.edu.tw (Y.-L.T.); 2 Faculty of Veterinary Medicine, Rajamangala University of Technology Tawan-ok, Chonburi 20110, Thailand; suttitas_to@rmutto.ac.th; 3 Research Center of Animal Biologics, National Pingtung University of Science and Technology, Pingtung 912301, Taiwan; 4 Department of Chinese Pharmaceutical Sciences and Chinese Medicine Resources, College of Chinese Medicine, China Medical University, 91 Hsueh-Shih Road, Taichung 40402, Taiwan

**Keywords:** chicken anemia virus, *Eimeria tenella*, concurrent co-infection, secondary infection

## Abstract

Chicken anemia virus (CAV) and *Eimeria tenella* (*E. tenella*) are economically important pathogens of the poultry industry worldwide. However, the impact of dual infection of these two pathogens in chickens remains unclear. This study investigated the pathogenic effects of dual infection with CAV and *E. tenella* using two trials. In Trial A, chickens were infected at 21 days of age (D21) with either CAV and *E. tenella* simultaneously (C_21_ + T_21_), CAV alone (C_21a_), *E. tenella* alone (T_21_), or PBS as a negative control (NC). In Trial B, chickens received CAV at D21 followed by *E. tenella* at D28 (C_21_ + T_28_), CAV alone at D21 (C_21b_), *E. tenella* alone at D28 (T_28_), or PBS at D21 (NC). Assays of lesion scores (LS), oocysts per gram (OPG) of feces, packed cell volume (PCV), and thymus index (TI) were used to assess variations in pathogenicity. Both the C_21_ + T_21_ and C_21_ + T_28_ groups showed higher OPG than the group infected with *E. tenella* alone, with significantly elevated OPG in the secondary infection scenario and more severe lesions in the concurrent co-infection group (*p* < 0.05). Anemia, indicated by PCV < 27%, was observed in the C_21_ + T_21_ group at day 28 and in the C_21_ + T_28_ group at day 35, both of which had significantly lower PCV values than the group infected with CAV alone (*p* < 0.001). Thymus atrophy was most severe in C_21_ + T_21_ at 28 days old (*p* < 0.05; *p* < 0.01). In this study, preliminary observations suggested that concurrent and secondary infections with CAV and *E. tenella* showed variable trends that may indicate potential interactions; however, these exploratory findings require more systematic validation in older chickens.

## 1. Introduction

Chicken anemia virus (CAV), a member of the *Anelloviridae* genus *Gyrovirus*, was first isolated in Japan [1] and has since become globally endemic. The virus primarily affects chicken through age-dependent mechanisms. In chickens under 2 weeks of age, CAV targets hematopoietic progenitor cells in bone marrow and T lymphocyte precursors in the thymus cortex [2], resulting in anemia and generalized lymphoid atrophy [3]. However, older chickens (over 3 weeks of age) may not display the typical clinical signs of chicken infectious anemia (CIA) [3], whereas their immune competence can be compromised, rendering them more vulnerable to co-infections, secondary infections, or even multiple infection by various pathogens [3].

To date, numerous studies have explored the interaction between CAV and other pathogens [4,5,6]. Co-infection with *Staphylococcus aureus* has been linked to bacterial chondronecrosis and osteomyelitis [4]. Concurrent infection with *Escherichia coli* exacerbates respiratory symptoms and increases mortality, while co-infection with *Clostridium perfringens* can cause hemorrhagic necrotic dermatitis affecting the wings, back, breast, and thighs, along with septicemic lesions in the bone marrow and internal organs [5]. Concurrent infection with *Escherichia coli* exacerbates respiratory symptoms and increases mortality [6]. These outcomes are mainly attributed to CAV-induced immunosuppression, which enhances bacterial invasion and disease severity, ultimately raising treatment costs and reducing chicken production performance.

Co-infection of CAV with various viral pathogens, including infectious bronchitis virus (IBV) [7], infectious bursal disease virus (IBDV) [8], Marek’s disease virus (MDV) [9], avian leukosis virus subgroup J (ALV-J) [10], fowl adenovirus serotype E8b (FAdV E8b) [11], and avian reovirus (ARV) [12], has been shown to remarkably worsen disease outcomes in chickens. These dual infections are associated with more severe clinical signs, increased mortality, and pronounced immunosuppression. The underlying mechanisms involve T cell depletion [7,8,9,10,11,12], reduced virus-specific antibody production [8], enhancement of the cytolytic phase of virulent MDV strains [9], and interference with immune organ recovery [9]. In particular, co-infection with FAdV E8b resulted in abnormal biochemical markers and mortality rates reaching 100 percent [11], while co-infection with ARV led to reduced weight gain and more severe anemia [12]. These findings highlight the role of CAV in amplifying the pathogenicity of a broad range of viral agents.

In addition, co-infection of CAV with protozoan pathogens has been reported to exacerbate clinical and pathological effects. Infections involving *Leucocytozoon caulleryi* (*L. caulleryi*) showed that CAV intensified vascular endothelial damage, leading to widespread hemorrhage in multiple organs and more severe anemia due to enhanced erythrocyte destruction [13]. Co-infection with *Plasmodium juxtanucleare* (*P. juxtanucleare*) in specific-pathogen-free White Leghorn chickens resulted in moderate organ enlargement and significantly higher parasitemia [14]. Furthermore, concurrent infection with *Cryptosporidium baileyi* (*C. baileyi*) increased oocyst shedding and led to their presence in atypical locations within the bursa of Fabricius [15]. These results further support the role of CAV in promoting disease severity through immune modulation across different types of pathogens.

Chicken coccidiosis caused by apicomplexan protozoan of genus *Eimeria* represents a major global veterinary health challenge in poultry production. This disease causes annual losses of USD 13 billion through production impacts and control costs [16]. Seven established species (*E. acervulina*, *E. brunetti*, *E. maxima*, *E. necatrix*, *E. praecox*, *E. mitis*, and *E. tenella*) and three recently discovered species (*E. lata*, *E. nagambie*, and *E. zaria*) affecting chickens have been reported [17,18]. Among them, *E. tenella* emerges as one of the most pathogenic *Eimeria* species [19]. The main pathogenic progression of this disease involves the second generation of merozoites causing extensive destruction of epithelium and capillaries in ceca, resulting in hemorrhage and dysentery [20,21,22]. Severe infection induces necrosis and atrophy of the cecal villi, leading to a reduced feed conversion ratio (FCR), growth retardation, and increased mortality [23]. In addition, due to the mucosal barrier function disruption, it facilitates the proliferation of *Clostridium perfringens* [24] and promoting colonization by other enteric pathogens including *Campylobacter jejuni* [19,25], *Escherichia coli*, and *Salmonella* spp. [24,26,27]. This intestinal dysbiosis alters host metabolism and microbiome equilibrium, intensifying disease pathogenesis and necessitating therapeutic approaches [26]. Beyond co-infection between *E. tenella* and other pathogens, mixed-species infections involving different *Eimeria* species are also common in commercial chicken farms [28,29]. Under experimental infection conditions, the extent to which these interactions exacerbate pathogenicity depends on the specific combinations (e.g., *E. mitis* with *E. tenella*, or *E. mitis* with *E. necatrix*) and the evaluated parameters, such as mortality, intestinal lesions, oocyst output, etc. [30].

Chicken anemia virus is capable of both vertical and horizontal transmission, and chickens exhibit high susceptibility to infection even on the first day post-hatching [3]. In contrast, *E. tenella* is not transmitted vertically, and clinical disease associated with this pathogen is more frequently observed in chickens older than three weeks [3]. However, CAV and *E. tenella* are highly resistant to environmental factors and are therefore commonly spread in chicken farms worldwide [31,32]. Based on our clinical diagnostic experience, dual infections with CAV and *E. tenella* are frequently observed in older pullets (≥3 weeks of age), with each pathogen presenting its distinct characteristic symptoms. Despite the administration of high doses of anticoccidial agents and prolonged treatment durations, farmers consistently report difficulties in effectively controlling the disease.

Based on previous findings, many studies have investigated co-infections of either CAV or *E. tenella* with other pathogens in chickens and revealed potential synergistic effects. However, few have examined the direct interaction between CAV and *E. tenella*. The pathogenic variations resulting from their dual infection, as well as the impact of the temporal sequence of infection, remain unclear to date. Therefore, the purpose of this study is to investigate whether the interaction between CAV and *E. tenella* is associated with a synergistic enhancement of pathogenicity in the host. Cecal lesion scores (LS), oocysts per gram (OPG) of feces, packed cell volume (PCV), and thymus index (TI) were employed to assess the effects of concurrent co-infection and secondary *E. tenella* infection introduced one week after CAV.

## 2. Materials and Methods

### 2.1. Chickens and Housing

Ninety-six 21-day-old specific-pathogen-free (SPF) Leghorn chickens (JD-SPF Biotech Co., Ltd., Miaoli, Taiwan) were housed in filtered-air isolators with group separation for biosecurity. Animals had unrestricted access to feed and water, both verified to be free of anticoccidial agents throughout the experimental period. All experimental procedures were conducted in accordance with protocols that had been reviewed and approved by the Institutional Animal Care and Use Committee of National Pingtung University of Science and Technology, Neipu, Taiwan (IACUC; NPUST-108-067. Approval date: 5 February 2020).

### 2.2. CAV Propagation and TCID_50_ Determination

A field isolate of CAV (1705PT; GenBank Accession No. MK386570), obtained in 2017 from 3-week-old chickens in Taiwan exhibiting typical signs of infectious anemia, was selected for this study. The virus was propagated in Marek’s disease chicken cell (MDCC)-MSB1 cells, provided by Dr. Meng-Shiou Lee (China Medical University, Taichung, Taiwan). MDCC-MSB1 cells were maintained in RPMI 1640 medium with 10% fetal bovine serum (FBS) (Gibco, Grand Island, NY, USA) and 1% antibiotic–antimycotic solution (Gibco, Grand Island, NY, USA) at 37 °C in a 5% CO_2_ humidified incubator (Forma Steri-Cycle, Thermo Fisher Scientific, Cincinnati, OH, USA). The infected cultures were incubated for 72 h, after which the virus-containing supernatant was harvested, clarified by centrifugation at 2000× *g* for 10 min, and stored at −80 °C until further use. MDCC-MSB1 cells were plated in 96-well plates (1.0 × 10^5^ cells/well) for tissue culture infectious dose 50 (TCID_50_) determination. Ten-fold serial dilutions of the virus stock (100 μL/well) were added to eight replicate wells, and the plates were incubated at 37 °C for 3 days, with cytopathic effect (CPE) monitored daily and TCID_50_ calculated using the Reed–Muench method [33,34].

### 2.3. Isolation and Propagation of E. tenella

The *E. tenella* wild-type strain (GenBank Accession No. MN252103) used in this study was derived from a single oocyst and purified according to modified protocols [20,35]. Strain propagation was achieved through oral inoculation of coccidia-free chickens 6 days post-infection. Fecal samples were homogenized, mixed with saturated sodium chloride solution, and centrifuged. The resulting supernatant was collected, washed twice with double-distilled water, and subjected to a final centrifugation (1200× *g*, 2 min) to precipitate oocysts. Oocysts completed sporulation in 2.5% (*w*/*v*) potassium dichromate solution under gentle agitation at ambient temperature for 72 h. Complete sporulation (>90%) was confirmed by clearly observing 4 sporocysts within each oocyst. The sporulated oocysts were subsequently maintained at 4 °C for experimental use.

### 2.4. Experimental Design

#### 2.4.1. Trial A: Concurrent Co-Infection

A total of 48 chickens were randomly assigned to four groups (*n* = 12 per group). Among these, chickens in the three treatment groups were infected at 21 days of age (D21) with either both CAV and *E. tenella* simultaneously (C_21_ + T_21_), CAV alone (C_21a_), or *E. tenella* alone (T_21_). The infection doses were 1.0 × 10^4.5^ TCID_50_/bird for CAV and 2.5 × 10^4^ sporulated oocysts/bird for *E. tenella*. The negative control (NC) group received phosphate-buffered saline (PBS) at D21.

**Figure 1:**
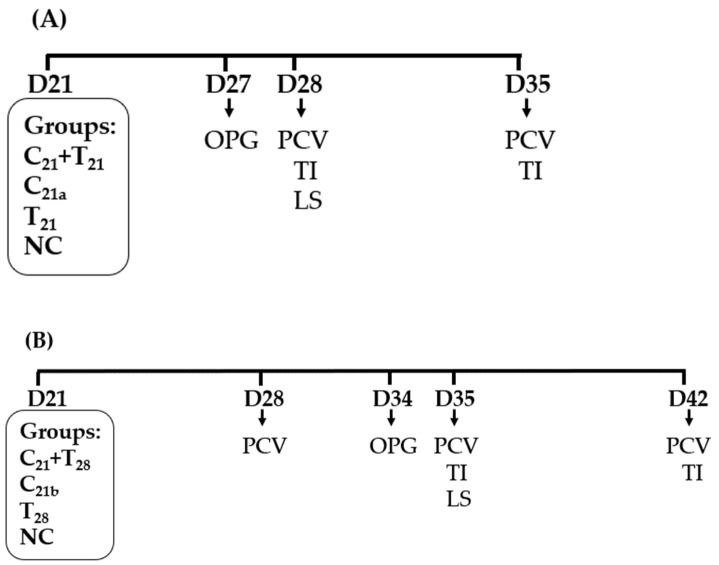
Schematic presentation of chicken anemia virus (CAV) and/or *Eimeria tenella* (*E. tenella*) infections and sampling. (**A**) In Trial A, all treatment groups were infected with CAV and/or *E. tenella* on day 21 (D21). The negative control (NC) group received phosphate-buffered saline (PBS) only. Concurrent co-infection with CAV and *E. tenella* was designated as C_21_ + T_21_, infection with CAV alone as C_21_a, and with *E. tenella* alone as T_21_. (**B**) In Trial B, chickens were infected with CAV at D21 and secondarily with *E. tenella* at D28 (C_21_ + T_28_). Groups infected with CAV alone at D21 or *E. tenella* alone at D28 were designated as C_21b_ and T_28_, respectively. Downward arrows indicate sampling for ceca lesion scores (LS), oocysts per gram (OPG) of feces, packed cell volume (PCV), and thymus index (TI).

At D27, 12 chickens in each group were divided into four subgroups, each containing three chickens. Fecal samples were then collected to quantify OPG (*n* = 4). At D28, blood samples were collected from each bird to determine PCV values, and five chickens in each group (5/12) were randomly selected and then euthanized by one-time electrical stunning for the assessment of LS in ceca and TI in thymus. At D35, the remaining seven chickens were sampled for PCV values, and 5 of the chickens were randomly selected and sacrificed for TI evaluation (Figure 1A).

#### 2.4.2. Trial B: Secondary *E. tenella* Infection

Except for the timings for pathogen infection and sampling, the number of chickens used, and the number of groups, infection doses, and assays conducted in Trial B were similar to those in Trial A. As shown in Figure 1B, a treatment group was infected with CAV at D21 and secondarily infected with *E. tenella* at D28, defined as C_21_ + T_28_. The other two treatment groups were, respectively, infected with CAV at D21 and *E. tenella* at D28. The NC group received PBS at D21.

At D28, blood samples were collected from each bird to determine PCV values. At D34, 12 chickens in each group were divided into four subgroups, each containing three chickens. Fecal samples were then collected to calculate OPG (*n* = 4). At D35, blood samples were collected from each bird to determine PCV values, and five chickens in each group (5/12) were randomly selected and then euthanized by one-time electrical stunning for the assessment of LS in ceca and TI in thymus. At D42, the remaining seven chickens were sampled for PCV values, and 5 of the chickens were randomly selected and sacrificed for TI evaluation.

### 2.5. Ceca Lesion Assessment

After chickens were euthanized by one-time electrical stunning, the ceca were immediately examined for gross pathology by experienced operators and lesions were scored according to the methodology established by Johnson and Reid [36]. Ceca lesions were rated on a scale from 0 to 4, where 0 indicates no lesions and 4 signifies severe lesions. Mortality was recorded, with dead chickens assigned a score of 4.

### 2.6. Fecal Oocyst Output

The OPG counting procedure was performed with slight modifications according to the protocols as previously described [20,37]. Briefly, 5 g fecal samples were homogenized with 15 mL of saturated saline solution for oocyst flotation, and the resulting suspension was examined in triplicate using the McMaster counting technique under a compound microscope (Nikon E-400, Tokyo, Japan; 10× objective).

### 2.7. Hematocrit Determination

Blood samples were collected from the wing vein into heparinized microhematocrit capillary tubes. The capillary tubes were sealed at one end with clay and centrifuged at 1200× *g* using an HCD-2000 centrifuge (Chuanhua Precision Corp., New Taipei City, Taiwan) for 5 min to separate the cellular components from the plasma. Packed cell volume was measured with a standardized hematocrit reader card. Values are given as the percentage of red blood cells in total blood volume. Chickens with PCV < 27% were classified as anemic, following established poultry hematology standards as previously described [38].

### 2.8. Thymus Weight to Body Weight Ratio

The TI assay utilized a modified version of an established protocol [39]. Each chicken was weighed on a calibrated scale, then humanely euthanized. The thymus was excised using sterile instruments, and all visible fat was removed to ensure accurate weight measurement. The cleaned thymus was weighed on a high-precision balance (±0.1 mg), with measurements recorded immediately to prevent dehydration-related weight loss. The TI was determined by calculating the ratio of thymus weight to body weight (mg/g), offering a standardized measure for evaluating immune function.

### 2.9. Statistical Analysis

Statistical analyses were conducted using IBM SPSS Statistics ver. 27 (IBM Co., Armonk, NY, USA). For continuous variables, OPG, PCV, and TI, if the parameters obtained were normally distributed, statistical analysis was conducted using two-sample *t* test, or one-way ANOVA followed by Tukey’s post hoc test; if the parameters were not normally distributed, the Mann–Whitney U test or Kruskal–Wallis test followed by Dunn’s post hoc test was applied. For ordinal variable, LS, the Mann–Whitney U test was applied. Statistical significance is denoted by asterisks as * (*p* < 0.05), ** (*p* < 0.01), and *** (*p* < 0.001), corresponding to significant, very significant, and highly significant differences, respectively.

## 3. Results

### 3.1. Potential Ceca Lesion Variability in CAV and E. tenella Co-Infected Groups

**Figure 2:**
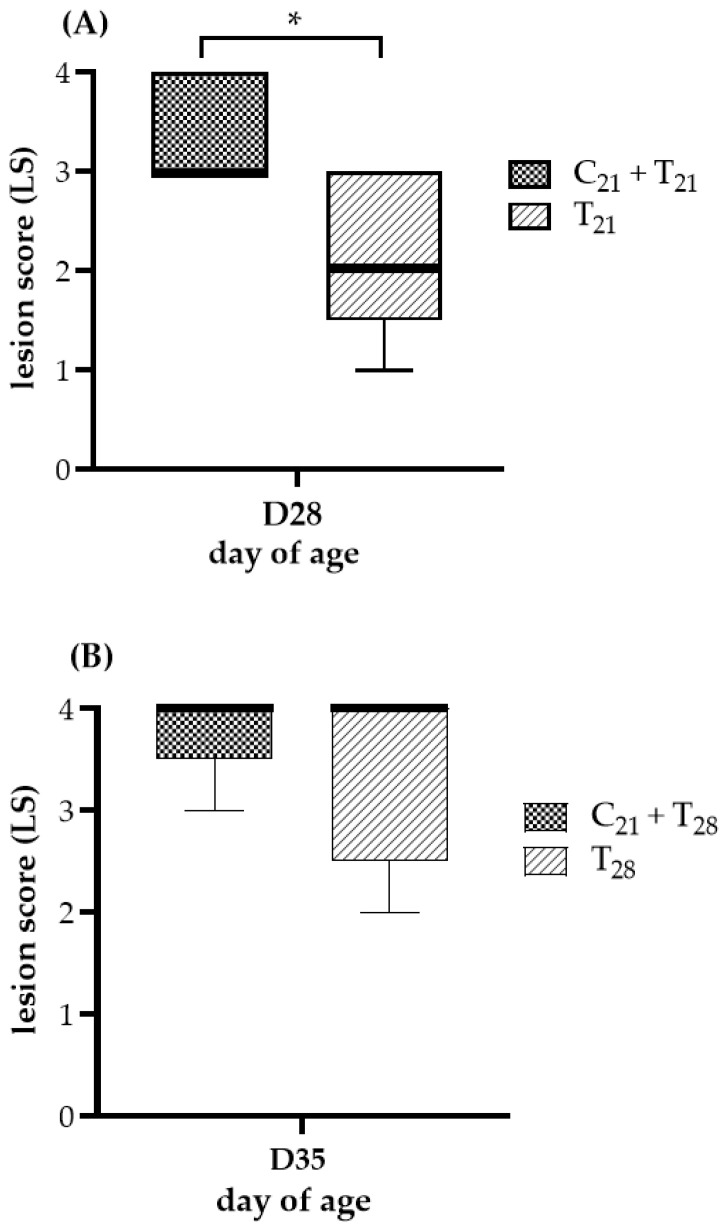
Ceca lesion severity after dual infection with chicken anemia virus (CAV) and *Eimeria tenella* (*E. tenella*) vs. *E. tenella* alone. (**A**) Trial A: Lesions were evaluated on Day 28 (7 days post-infection, dpi, with *E. tenella*), comparing concurrent co-infection with CAV and *E. tenella* at 21 days of age (C_21_ + T_21_) vs. *E. tenella* infection alone at the same age (T_21_). (**B**) Trial B: Lesions were evaluated on Day 35 (7 dpi with *E. tenella*), comparing chickens infected with CAV at Day 21 and secondarily with *E. tenella* at Day 28 (C_21_ + T_28_) vs. *E. tenella* infection alone at Day 28 (T_28_). Five chickens per group (*n* = 5) were randomly selected for necropsy. Lesion severity was scored on a scale from 0 (normal) to 4 (most severe), following the criteria described by Johnson and Reid [36]. Medians and interquartile ranges (IQR) were compared using the Mann–Whitney U test and are shown as box plots. Asterisk (*) indicates *p* < 0.05.

The ceca lesion scores at 7 days post-infection with *E. tenella* are shown in Figure 2. No lesions were observed in chickens from the negative control group or the group infected with CAV alone, resulting in a score of 0, indicating that no *E. tenella* contamination occurred. In Trial A, a comparison using the Mann–Whitney test showed that the median lesion score in the C_21_ + T_21_ group was significantly higher than that in the T_21_ group (3 vs. 2; *p* = 0.033). In Trial B, the median lesion scores of the C_21_ + T_28_ and T_28_ groups were identical (4 vs. 4), with no statistically significant difference (*p* = 0.44). No chickens died throughout the entire experiment; therefore, none were assigned a lesion score of 4 due to *E. tenella* infection. Overall, under concurrent co-infection scenarios, this suggests that CAV may exacerbate the ceca damage caused by *E. tenella*.

### 3.2. Oocyst Counts Observed in Dual Infection with CAV and E. tenella

**Figure 3:**
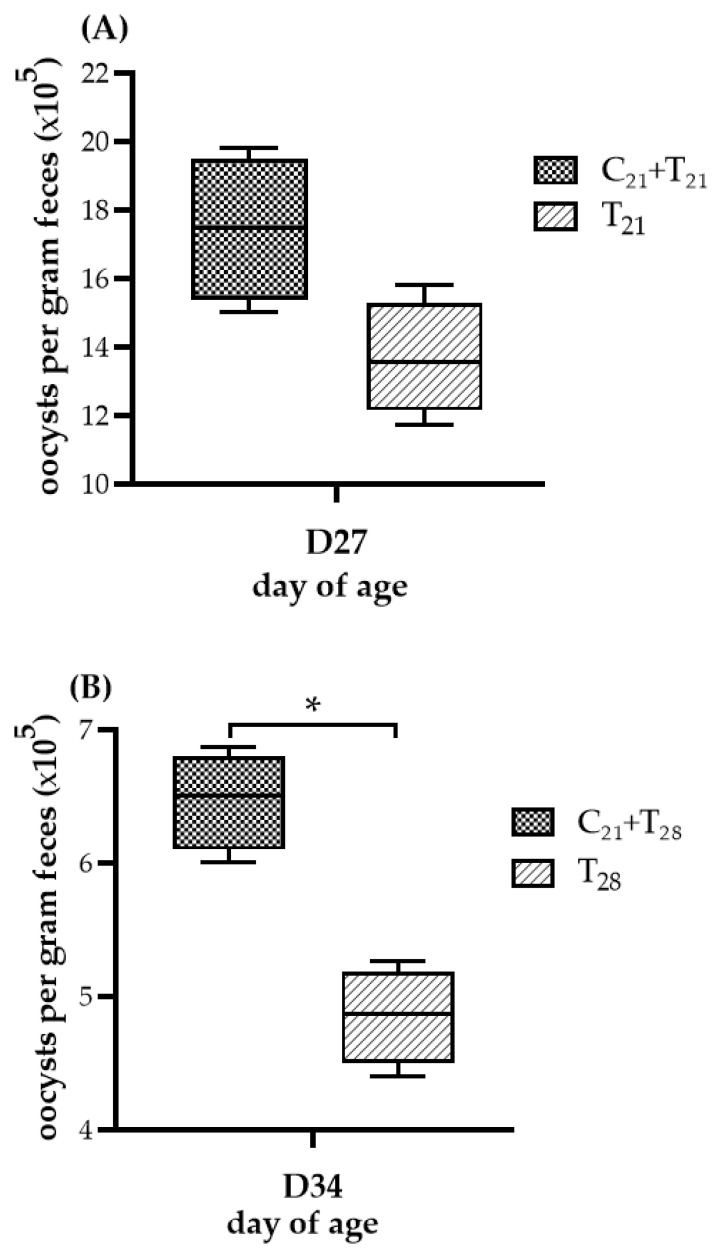
Oocyst shedding following dual infection with chicken anemia virus (CAV) and *Eimeria tenella* (*E. tenella*) vs. *E. tenella* alone. (**A**) Trial A: Oocysts per gram (OPG) of feces measured on Day 27 (5 days post-infection, dpi, with *E. tenella*), comparing the concurrent infection group at Day 21 (C_21_ + T_21_) vs. the group infected with *E. tenella* alone (T_21_). (**B**) Trial B: OPG measured on Day 34 (5 dpi with *E. tenella*), comparing secondary infection group (C_21_ + T_28_) vs. the group infected with *E. tenella* alone (T_28_). Each group (*n* = 12) was divided into 4 subgroups; pooled feces from 3 chickens per subgroup (*n* = 4) were analyzed using the McMaster counting method. Results ( × 10^5^) are shown as box plots with medians and interquartile ranges (IQR) using Mann–Whitney U test. Asterisk (*) indicates *p* < 0.05.

The OPG of chickens given the different treatments is presented in Figure 3. No oocysts were observed in chickens from the negative control group and the group infected with CAV alone, indicating that no *E. tenella* contamination occurred. While both trials showed comparable patterns in OPG levels between the dual-infected and solely *E. tenella*-infected groups, the statistical significance differed. In Trial A, the median OPG value in the T_21_ group was higher than that in the C_21_ + T_21_ group, but the difference was not statistically significant (median 1.75 × 10^6^ vs. 1.36 × 10^6^; *p* = 0.057). In contrast, in Trial B, the C_21_ + T_28_ group showed a significantly higher median OPG than the T_28_ group (median 6.50 × 10^5^ vs. 4.87 × 10^5^; *p* = 0.021). These findings suggest that prior infection with CAV may enhance oocyst proliferation during subsequent *E. tenella* infection in the ceca.

### 3.3. Potential Interactions of Concurrent and Secondary Infection on Severity of Anemia

**Figure 4:**
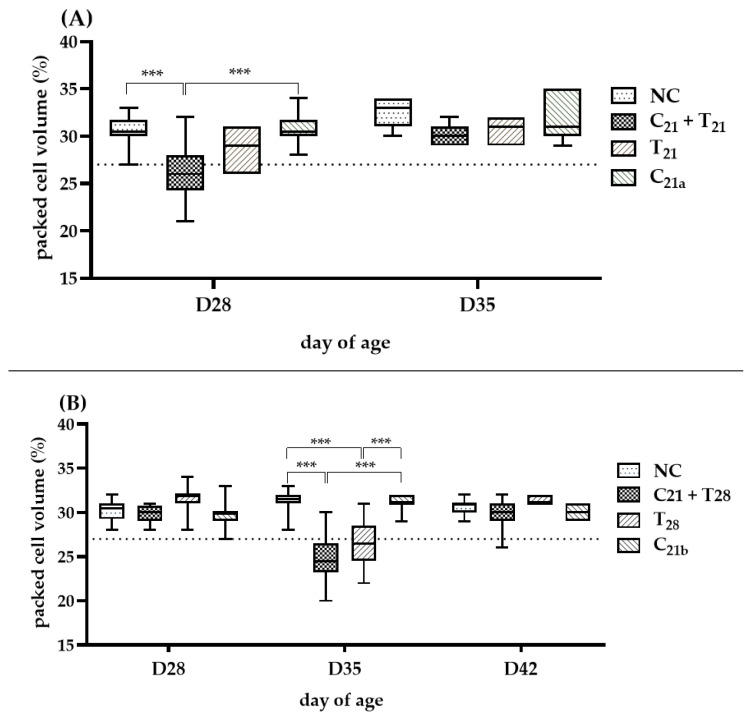
Assessment of anemia induced by chicken anemia virus (CAV) and/or *Eimeria tenella* (*E. tenella*). In Trial (**A**), packed cell volume (PCV) was measured in 12 chickens per group on Day 28 (7 days post-infection, dpi, with CAV and/or *E. tenella*), and in 7 chickens on Day 35 (*n* = 7; 14 dpi). In Trial (**B**), PCV was measured in 12 chickens per group on Day 28 (7 dpi with CAV) and Day 35 (14 dpi with CAV; 7 dpi with *E. tenella*), and in 7 chickens on Day 42 (*n* = 7; 21 dpi with CAV; 14 dpi with *E. tenella*). Statistical analysis was performed using the Kruskal–Wallis test followed by Dunn’s post hoc test. Box plots show medians and interquartile ranges (IQR). The dashed line represents the anemia threshold (<27%). Asterisks (***) indicate *p* < 0.001.

The severity of anemia (PCV < 27%) in each group is shown in Figure 4. In Trial A (D28), the C_21_ + T_21_ group developed anemia in 7 of 12 birds (median 26%), significantly more severe than in the C_21a_ group (4/12; median 30.5%, *p* < 0.001). In Trial B (D35), the C_21_ + T_28_ group showed anemia (median 24.5%), significantly lower than the C_21b_ group (median 31%; *p* <0.001). Although the median PCV in the T_28_ group was only slightly below the anemia threshold (27%), it still indicated anemia (median: 26.5%). However, the number of anemic birds was higher in the C_21_ + T_28_ group than in the T_28_ group (9/12 vs. 6/12 birds). At other time points, such as Day 35 in Trial A and Days 28 and 42 in Trial B, the median PCV values remained above the 27%, and thus no clear anemia trend could be established for comparison.

Overall, in concurrent co-infection scenarios, CAV-associated anemia tended to be more pronounced at Day 28 following the addition of *E. tenella*, whereas in secondary infection scenarios, this effect was more noticeable at Day 35.

### 3.4. Variable Thymus Atrophy Under Dual Infection with CAV and E. tenella

**Figure 5:**
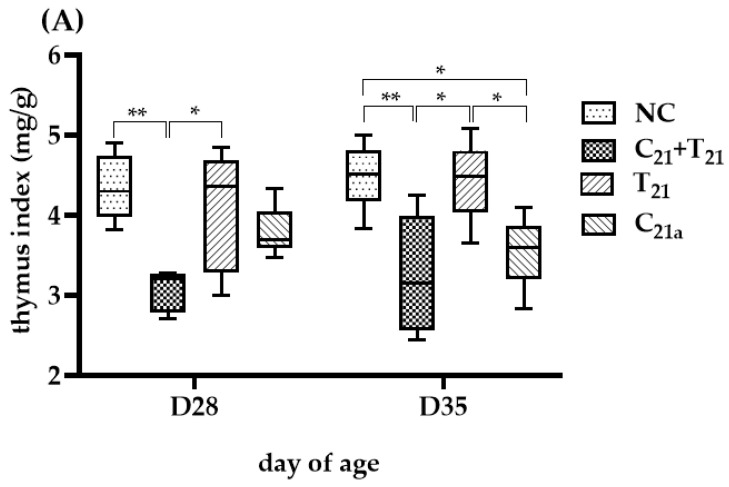

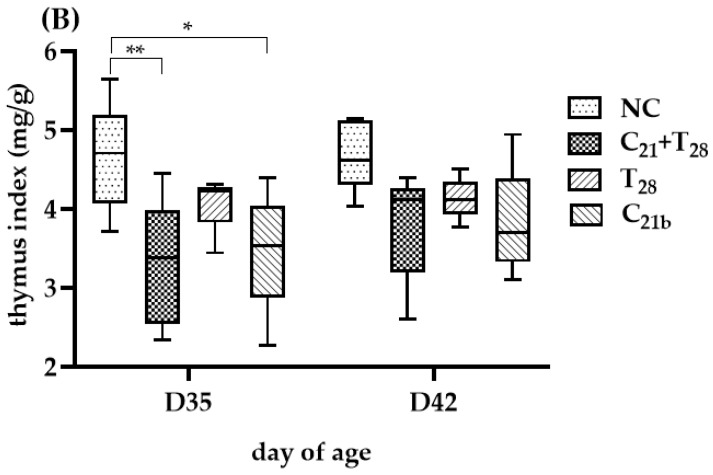
Thymus atrophy was evaluated using the thymus index (TI), calculated as the ratio of thymus weight to body weight (mg/g), which is inversely related to the degree of atrophy. In Trial (**A**), TI values were obtained from 5 chickens per group on Day 28 (7 days post-infection [dpi] with CAV and/or *E. tenella*; *n* = 5), and from another 5 chickens on Day 35 (14 dpi; *n* = 5). In Trial (**B**), 5 chickens per group were sampled on Day 35 (14 dpi with CAV; 7 dpi with *E. tenella*; *n* = 5) and Day 42 (21 dpi with CAV; 14 dpi with *E. tenella*; *n* = 5). Statistical analysis was conducted using the Kruskal–Wallis test followed by Dunn’s post hoc test. Box plots display medians and interquartile ranges (IQR). Asterisks indicate significance (*, *p* < 0.05, and **, *p* < 0.01).

The degree of thymus atrophy, indicated by TI values, is shown in Figure 5. On D35, the groups infected with CAV alone had significantly lower median TI values than the negative controls (3.6 vs. 4.51, *p* = 0.01 in Trial A; 3.54 vs. 4.71, *p =* 0.02 in Trial B), while the groups infected with *E. tenella* alone showed similar values to controls with no statistical significances (4.49 vs. 4.51, *p* = 0.75; 4.23 vs. 4.71, *p =* 0.13), indicating CAV as the main cause of atrophy. Furthermore, TI values in the C_21_ + T_21_ and C_21_ + T_28_ groups were significantly lower than their respective controls (3.15 vs. 4.51, *p =* 0.007; 3.38 vs. 4.71, *p* = 0.045), and also lower than the groups infected with CAV alone (C_21a_ and C_21b)_, though not significantly (3.15 vs. 3.6, *p =* 0.83; 3.54 vs. 4.71, *p* = 0.75). Compared to the T_21_ and T_28_ groups (3.15 vs. 4.49; 3.38 vs. 4.23), the difference was significant in Trial A (*p* = 0.01), but not in Trial B (*p* = 0.23). These findings suggest that concurrent co-infection and secondary *E. tenella* infection may aggravate thymus atrophy at this time point.

At D28, TI values in the C_21a_ and T_28_ groups were similar to controls (3.69 vs. 4.3, *p =* 0.18; 4.36 vs. 4.3, *p =* 0.45), while the C_21_ + T_21_ group showed a significantly lower TI than the control (3.21 vs. 4.3, *p* =0.002), but not different from the C_21a_ group (3.21 vs. 3.69, *p* =0.07), indicating a potential supportive role of *E. tenella* in CAV-induced atrophy.

## 4. Discussion

The term co-infection is frequently used in studies of host–pathogen interactions; however, its definition can be somewhat variable. Based on a study of synergistic or antagonistic interactions during co-infections with homologous or heterologous pathogens in fish, Kotob et al. (2016) defined it as the simultaneous or sequential infection of a single host by two or more distinct pathogens that remain active within the host [40]. Other terms such as dual infections, multiple infections, mixed infections, complicated infections, super infections, concurrent infections, and secondary infections are sometimes regarded as variants or subtypes of co-infection [41]. However, with the exception of concurrent and secondary infections, these terms generally do not imply a specific timing or sequence of infection [42]. Karvonen et al. (2019) [42] further noted that co-infections often occur sequentially, with time intervals between infections that can markedly influence disease outcomes. Similarly, Bakaletz (2004) suggested that pathogen interactions can influence disease severity or vaccine efficacy, and that animal models are often needed to confirm these effects [41]. These insights support our investigation of how infection timing and sequence influence pathogenicity. Thus, we use the terms concurrent, secondary, and dual infection to better reflect our experimental design.

Regarding pathogen propagation systems, *E. tenella* is adaptable to both Madin–Darby bovine kidney (MDBK) cells and chicken embryos [3], whereas CAV has only been successfully propagated in MDCC MSB-1 cells and not in chicken embryos [3]. Consequently, animal experiments were necessary in this study, as live chickens are the only known host system capable of supporting both pathogens, enabling the examination of their potential interactions. Since infection with CAV alone does not induce pathological symptoms in the ceca or result in oocyst shedding, lesion scores and oocyst counts per gram of feces are commonly used as specific indicators of *E. tenella* pathogenicity. Therefore, observations of LS and OPG dynamics may help to elucidate whether and how CAV affects the disease outcome of *E. tenella* infection.

In this exploratory study, trends were observed suggesting that both the concurrent co-infection group (C21 + T21) and secondary *E. tenella* infection groups (C21 + T28) tended to show higher OPG than the groups infected with *E. tenella* alone, and the C21 + T21 group appeared to exhibit more severe lesions in ceca. While these observations suggest potential interactions, the limited sample size and high variability prevent definitive conclusions about pathogenic synergism. These preliminary findings show some consistency with a previous report showing that co-infection with CAV and *C. baileyi* occurs in pathogenic variants [43]. *E. tenella*, like *C. baileyi*, is a protozoan belonging to the order Eucoccidiorida and exhibits a comparable life cycle [23]. The trends observed during dual infection with CAV and *E. tenella* may be analogous to those reported with CAV and *C. baileyi*, though further validation is needed. Additionally, Abou EL-Azm et al. (2022) observed that co-infection with CAV and *Leucocytozoon caulleryi* led to increased severity of pathology [13]. This provides some context for our preliminary observations of increased oocyst shedding and ceca lesions in dual infections, though direct comparisons require caution given methodological differences. Similar patterns have been reported with infectious bursal disease virus (IBDV) and *E. tenella* co-infections [44], suggesting that interactions between immunosuppressive agents and *Eimeria* spp. may represent a broader phenomenon worthy of systematic investigation.

In this exploratory study, preliminary observations suggested that chickens in the dual infection groups appeared to show reduced activity and appetite compared to those infected with *E. tenella* alone, though these behavioral changes were not systematically quantified. While LS and OPG represent the most practical indicators for assessing coccidian pathogenicity [23], our results across these parameters and infection timing showed considerable inconsistency and high variability. Given that lesion scores and oocyst counts reflect different biological processes influenced by parasite life cycle, host immunity, and treatment [23], and considering the complex relationship where severe lesions may occur with low oocyst output and vice versa [42], the interpretation of our limited dataset requires substantial caution. The lack of statistical robustness, small sample sizes, and inconsistent patterns prevent definitive conclusions about pathogenic interactions. Therefore, our findings should be considered hypothesis-generating observations that suggest potential trends warranting systematic investigation rather than evidence of enhanced pathogenicity between CAV and *E. tenella*.

In clinical diagnosis of CAV infection in chickens, indicators such as anemia and thymus atrophy are commonly used. However, due to an age-resistance characteristic, anemia is typically observed only in young chickens [45]. A previous report indicated that in chickens older than 3 weeks, anemia was observed only when a high dose of CAV (10^6^ TCID_50_ / bird) was administered, whereas a low dose (10^3^ TCID_50_ / bird) did not cause anemia. In this study, we used a medium dose (1 x 10^4.5^ TCID_50_ / bird) as the infection dose. As expected, chickens infected with CAV alone did not exhibit anemia at any of the observed time points. In contrast, anemia was observed in both the concurrent co-infection and secondary groups following *E. tenella* infection (Figure 4A,B), suggesting a potential role of *E. tenella* in modulating the pathogenic outcome of CAV. However, at Day 35 (7 dpi with *E. tenella* and 14 dpi with CAV) in Trial B, mild anemia was also observed in the group infected with *E. tenella* alone, although fewer birds were affected compared to the secondary infection group (6/12 vs. 9/12). This may have resulted from severe hemorrhage in the ceca caused by *E. tenella*, which could confound the interpretation of CAV’s role in anemia development. Nonetheless, the findings raise the possibility that prior CAV infection may increase host susceptibility to *E. tenella*-associated ceca damage, thereby exacerbating blood loss and anemia. Conversely, *E. tenella* may also influence the course or severity of CAV-induced pathology, suggesting a potential bidirectional interaction between the two pathogens.

Thymus atrophy is a common pathological feature observed in chickens following infection with CAV. This condition primarily arises because CAV disrupts T lymphocyte precursors within the thymus cortex, with CD8^+^ cells being particularly susceptible to viral damage [9,46]. A study by Tongkamsai et al. (2019) reported that oral infection of 21-day-old chickens with a moderate dose of CAV (2 × 10^4.5^ TCID_50_/bird) resulted in thymus atrophy observed at D35 and D42. No atrophy was observed as early as D28 or as late as D49, with the latter likely reflecting recovery from the disease [39]. In the present study, a lower dose of CAV (1 × 10^4.5^ TCID_50_/bird) was used to reduce the severity of thymus atrophy and to allow clearer comparison with the dual infection groups.

In this study, chickens infected with CAV alone did not show thymus atrophy at Day 28 but did so by Day 35, while those infected with *E. tenella* alone showed no atrophy at any time point. In contrast, thymus atrophy was observed in all dual-infection groups, suggesting that the presence of *E. tenella* may enhance CAV-induced thymus damage, particularly at Day 28. By Day 35, this potential effect may have been masked by the atrophy already caused by CAV alone, as differences between groups were less apparent. In addition, although our results do not support a direct atrophic effect of *E. tenella*, this possibility cannot be entirely ruled out. One study has reported reduced thymus-to-body weight ratios in broilers co-infected with multiple *Eimeria* species [44], while others, based on experimental co-infection with different *Eimeria* strains, reported no significant change in thymus size [30]. One possible explanation involves the gut–thymus axis, in which microbial metabolites such as peptidoglycan and short-chain fatty acids influence thymus T cell development. Disruption of these signals, possibly through microbiota imbalance, may impair T cell differentiation and lead to thymus atrophy [46]. While this mechanism remains speculative, future studies incorporating immunological and molecular approaches such as cytokine profiling, quantitative real-time PCR (qPCR), lymphocyte dynamics, and histopathology are needed to clarify the role of *E. tenella* in thymus atrophy.

## 5. Conclusions

Chicken coccidiosis caused by *Eimeria* spp. is one of the most economically significant and difficult-to-control poultry diseases [23]. In contrast, CAV infection often remains undetected due to its subclinical nature, although it may contribute to immunosuppression, particularly in older birds [3]. This study aimed to explore potential interactions between these pathogens and their possible combined effects on host pathogenicity.

Preliminary findings from controlled infections with varying timing and order suggest that CAV may be associated with enhanced pathogenicity of *E. tenella*, and possibly vice versa. However, the extent and consistency of these effects appeared to vary across clinical indicators and experimental protocols. Some effects observed in this study were relatively modest, which may be partly attributed to the controlled conditions and the limited sample size.

In view of these limitations, the findings should be interpreted with caution. Nevertheless, they may provide a useful reference for future studies incorporating larger sample sizes and more detailed immunological assessments. Considering that mixed *Eimeria* infections are frequently observed in field settings [30], and that farm-level co-infections are often influenced by other pathogens and environmental stressors, further investigation into the dynamics of CAV and *Eimeria* co-infection appears to be warranted for improving disease prevention and management strategies.

## Data Availability

The original contributions presented in this study are included in the article. Further inquiries can be directed to the corresponding authors.
